# Age-Related Decline in Anticipatory Motor Planning and Its Relation to Cognitive and Motor Skill Proficiency

**DOI:** 10.3389/fnagi.2017.00283

**Published:** 2017-09-04

**Authors:** Tino Stöckel, Kathrin Wunsch, Charmayne M. L. Hughes

**Affiliations:** ^1^Sport and Exercise Psychology Unit, Department of Sport Science, University of Rostock Rostock, Germany; ^2^Sport Psychology Unit, Department of Sport Science, University of Freiburg Freiburg, Germany; ^3^Department of Kinesiology, San Francisco State University, San Francisco CA, United States; ^4^Health Equity Institute, San Francisco CA, United States

**Keywords:** grasp posture planning, aging, executive functions, cognition, motor performance

## Abstract

Anticipatory motor planning abilities mature as children grow older, develop throughout childhood and are likely to be stable till the late sixties. In the seventh decade of life, motor planning performance dramatically declines, with anticipatory motor planning abilities falling to levels of those exhibited by children. At present, the processes enabling successful anticipatory motor planning in general, as do the cognitive processes mediating these age-related changes, remain elusive. Thus, the aim of the present study was (a) to identify cognitive and motor functions that are most affected by normal aging and (b) to elucidate key (cognitive and motor) factors that are critical for successful motor planning performance in young (*n* = 40, mean age = 23.1 ± 2.6 years) and older adults (*n* = 37, mean age = 73.5 ± 7.1 years). Results indicate that normal aging is associated with a marked decline in all aspects of cognitive and motor functioning tested. However, age-related declines were more apparent for fine motor dexterity, processing speed and cognitive flexibility. Furthermore, up to 64% of the variance in motor planning performance across age groups could be explained by the cognitive functions processing speed, response planning and cognitive flexibility. It can be postulated that anticipatory motor planning abilities are strongly influenced by cognitive control processes, which seem to be key mechanisms to compensate for age-related decline. These findings support the general therapeutic and preventive value of cognitive-motor training programs to reduce adverse effects associated with high age.

## Introduction

A characteristic of successful motor performance is the ability to plan and execute movements in such a fashion that everyday tasks can be accomplished. There are a number of factors that influence how actions are planned and executed, and the task of the individual is to optimize their performance within imposed task constraints (Newell, 1985). In that regard, most everyday life activities (e.g., grasping, walking, driving a car, etc.) rely on a person’s ability to appropriately plan movements prior to their initiation thereby considering situational constraints and future actions. For example, there is strong evidence that the hand postures used to grasp objects are particularly sensitive to future actions and task goals (cf. Rosenbaum et al., 2014). In their original study, Rosenbaum et al. (1990) asked participants to grasp a horizontally positioned bar and place it in a vertical position to either a left or right target. When the right side of the bar was to be placed to either target, all participants grasped the bar with an overhand grip. However, when the left side of the bar was to be placed to either target, participants always grasped the bar with an underhand grip. Thus, regardless of target location, in this condition participants always grasped the object in an awkward fashion, which ensured a comfortable hand posture at the end of the movement. Often called the end-state comfort effect, it provides evidence that people plan their movements to ensure comfortable final grasp postures in later stages of a movement, and that these postures are represented and planned prior to movement initiation.

There is strong evidence demonstrating that such anticipatory planning abilities improve as a function of age, reaching adult-like levels in late childhood to early adolescence (Stöckel et al., 2012; Stöckel and Hughes, 2015; see Wunsch et al., 2013 for a review). Across adolescence and adulthood anticipatory motor planning abilities remain quite stable until around 70 years of age, after which anticipatory planning proficiency rapidly declines to levels observed in young children (Scharoun et al., 2016; Wunsch et al., 2017). For example, in Wunsch et al. (2017) individuals between 60 and 70 (young-old group) and 71–80 years (old-old group) performed the traditional bar transport task and a version modified to test bimanual anticipatory motor planning. The authors found that there was a significant decrease in the preference for comfortable end postures for the old-old group (71- to 80-years-old) compared to the old-young group (60–70 years), and that planning performance of individuals in the old-old group was similar to that of children aged between 6 and 7 years. It was postulated that the decrease in anticipatory motor planning abilities in older adults was associated with declines in cognitive skills, a theory supported by empirical research demonstrating decreases in prefrontal cortex gray matter volume (Resnick et al., 2003; Raz et al., 2004), frontal and parietal white matter deterioration (Gunning-Dixon and Raz, 2003; Fazekas et al., 2005), and reduced levels of the neurotransmitters acetylcholine (Bartus et al., 1982; Gottfries, 1990), dopamine (Kaasinen and Rinne, 2002), serotonin (Gottfries, 1990), and norepinephrine (Marcyniuk et al., 1986).

Recently, researchers have sought to identify relationships between anticipatory motor planning and specific cognitive control processes, namely executive functions (Weigelt et al., 2009; Logan and Fischman, 2011; Stöckel et al., 2012; Stöckel and Hughes, 2016). Initial research by Weigelt et al. (2009) examined whether anticipatory motor planning is affected by memory demands during a sequential drawer opening task. In that study, participants opened a series of 11 drawers, with each drawer containing a cup with a written capital letter located on the bottom inside of the cup. Participants were instructed to open each drawer, look inside the cup to memorize the letter, place the cup back into the drawer, and then open the next drawer. After opening all of the drawers, participants had to recall as many letters as possible in the order that they were inspected (i.e., forward serial recall, Exp 1 & 2) or in any order they pleased (i.e., free recall, Exp 3). That study found that grasps were planned to maximize end-state comfort, but that normal memory performance (i.e., the ability to recall letters that appeared later in the movement sequence [the recency effect]) was disrupted. These results were taken as evidence that the motor demands of the task interfered with short-term memorization processes (e.g., the transfer of information to and/or the maintenance of information in short-term memory).

A recent study (Stöckel and Hughes, 2016) examined specific associations between three aspects of executive functioning (working memory, response planning and problem-solving, and inhibition) and two motor skill components (anticipatory motor planning and manual dexterity) in a sample of normally developing 5- and 6-year-olds. Results indicated that anticipatory motor planning performance was positively correlated with response planning and working memory capacity, whereas manual dexterity was correlated with working memory capacity and inhibitory control. Together, the existing evidence supports the idea that working memory and response planning are necessary executive functions required to successfully plan one’s motor actions. However, studies examining the relation between anticipatory motor planning and cognitive skills were conducted in developing (Stöckel et al., 2012; Stöckel and Hughes, 2016) and adult populations (Weigelt et al., 2009; Logan and Fischman, 2011), and as such to date there is no empirical evidence supporting the theory that age-related declines in anticipatory motor planning abilities are due to declines in executive functioning.

The purpose of the present study was hence to define key (cognitive and motor) factors that are critical for successful anticipatory motor planning performance in a sample of 77 neurologically and physically healthy young (*n* = 40, age range = 19–28 years) and older adults (*n* = 37, age range = 61–86 years). To address this issue, participants had to perform unimanual and bimanual bar transport tasks to assess individual’s motor planning skills, along with measuring the cognitive function components working memory (Corsi Block-Tapping Test [CBT], Backward Digit Span Test [DSpanbackward]), inhibitory control (Flanker Task, Simon Task), cognitive flexibility (Wisconsin Card Sorting Test [WCST], Trail Making Test [TMT]), planning and problem-solving abilities (Tower of London Task [TOL]), and processing speed (TMT-A) as well as the motor function components upper extremity flexibility (Back Scratch Test), gross and fine motor dexterity (Purdue Pegboard Test) and perceived grasp comfort (Grasp Comfort Ratings). Based on previous literature, it was hypothesized that anticipatory motor planning performance (Scharoun et al., 2016; Wunsch et al., 2017) as well as motor (Desrosiers et al., 1995, 1999; Daley and Spinks, 2000; Milanović et al., 2013) and cognitive skills (Levy, 1994; Park et al., 2003; Hedden and Gabrieli, 2004) would be significantly reduced in older adults as compared to their younger counterparts. Moreover, consistent with what was reported for pediatric populations (Stöckel and Hughes, 2016), it was expected that anticipatory motor planning would be associated with working memory and response planning in the older adult group. It has also been speculated that the tolerance for uncomfortable end postures is related to upper limb flexibility, such that the more limber an individual is the less likely it is to perceive an extreme joint angle as uncomfortable (Rosenbaum et al., 2014). If this hypothesis is correct, then anticipatory motor planning performance would be negatively associated with upper extremity flexibility and perceived comfort. However, based on what was reported for 5- and 6-year-old children (Stöckel and Hughes, 2016), anticipatory motor planning performance is not expected to be related to motor dexterity in general.

## Materials and Methods

### Participants

Forty independent community-dwelling older adults were recruited for this study, three of which withdrew from participation before any testing. This resulted in a sample of 37 older adults (age range = 61–86 years, mean age = 73.5 ± 7.1 years, mean handedness score = 0.79, 11 men). Forty healthy young adults (age range = 19–28 years, mean age = 23.1 ± 2.6 years, mean handedness score = 0.74, 18 men) served as a control group. All participants reported normal or corrected to normal vision, and did not have any known neuromuscular disorders. The study was approved by the institutional review board at the University of Rostock and conformed to the Declaration of Helsinki. Prior to participation, written informed consent was obtained from all participants.

### Measures and Procedures

To assess cognitive and motor function across this sample, the tests described below were administered to each participant. These tests were chosen because they provide a comprehensive assessment of cognitive and motor functions that are likely to constrain motor planning performance (cf. Rosenbaum et al., 2014; Stöckel and Hughes, 2016; Wunsch et al., 2017) using well-established standard tests that are appropriate for neurologically healthy individuals (i.e., no ceiling or floor effects for this age group). Each participant was assessed individually in a quiet room free from distraction. All participants were given breaks as and when necessary. The order of task administration was randomized across participants, with the exception of the comfort rating task that always followed the bar transport task. The entire experiment lasted approximately 90 to 120 min.

#### Motor Functions

**FIGURE 1:**
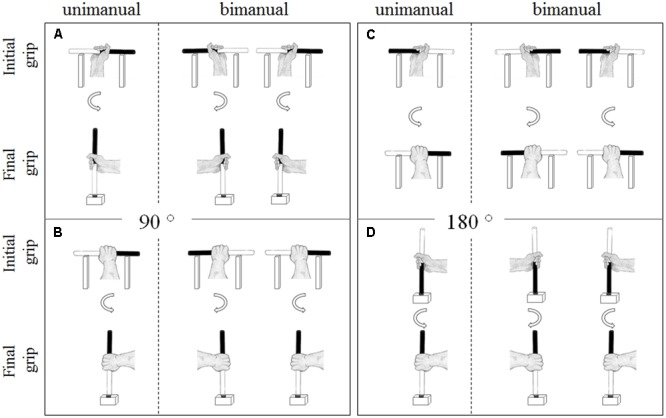
Bar-transport task. **(A)** Exemplar end-state comfort compliant grasp postures in unimanual and bimanual 90° (white end-down) conditions. **(B)** Exemplar end-state comfort non-compliant grasp postures in the unimanual and bimanual 90° (white end-down) conditions. **(C)** Exemplar end-state comfort compliant grasp postures in unimanual and bimanual 180° (horizontal bar position) conditions. **(D)** Exemplar end-state comfort non-compliant grasp postures in the unimanual and bimanual 180° (vertical bar position) conditions.

Anticipatory motor planning was assessed using the *unimanual and bimanual bar transport task* (Rosenbaum et al., 1990; Stöckel and Hughes, 2016; Wunsch et al., 2017; see **Figure 1**). In the 90° unimanual bar transport task, the to-be manipulated wooden cylinder (22 cm in height, 2 cm in diameter, painted black on one end and white on the other end) was horizontally positioned on a wooden cradle (20 cm in height, 20 cm length, with 10 cm between cradles). The target was a wooden cube (10 cm in height, 10 cm in length, 10 cm in width, with a 2.5 cm diameter hole in the center of the cube) located 10 cm in front of the cradle. In contrast, for the 180° unimanual bar transport task, the wooden cylinder was either positioned horizontally on the cradle or vertically in the target cube. At the start of each trial, the participant stood behind the starting line (90 cm away from the table) with their hands relaxed by their sides. After instructions specified which end of the cylinder (i.e., black or white) should be inserted into the target (or which end should point to the right), the participant walked up to the apparatus, picked up the cylinder with his preferred hand and inserted the required end into the target hole (or placing it on the cradle in the required orientation). After holding the bar at the target location for 5 s, the participant walked back to the starting position and the experimenter grasped the cylinder with a pincer grip and placed it back in preparation for the next trial. Instructions were standardized for all participants and identical to that used in previous studies (e.g., Stöckel et al., 2012; Stöckel and Hughes, 2016; Wunsch et al., 2017). Specifically, participants were informed that movement accuracy was of utmost importance and that they should perform the task at a comfortable speed, and no instructions were given about how to grasp the bar (i.e., overhand vs. underhand, thumb-up vs. thumb-down). Participants performed a total of 12 unimanual trials, comprised of two 90° end-orientation conditions (bar end orientation: black end to be inserted into the target, white end to be inserted into the target) and two 180° conditions (bar orientation: horizontally, vertically) with each condition performed three times. The start orientation of the bar (black end pointing to the left/ceiling, black end pointing to the right/floor) was counterbalanced across participants, and the individual trials were randomized. The percentage of trials that resulted in a comfortable thumb-up or palm-down posture at the end of the movement (end-state comfort satisfaction) was used as a measure of unimanual anticipatory motor planning (ESC_uni_).

During the bimanual task two experimental setups (i.e., cradles and cylinders) were placed on the table, standing 20 cm apart from each other (cf. Weigelt et al., 2006; Stöckel and Hughes, 2016; Wunsch et al., 2017). Participants reached for the cylinders resting on the cradles (in 90° and 180° bimanual conditions) or in the cubes (in the 180° bimanual condition) with both hands simultaneously and placed the instructed ends into the targets. The instructions were identical to the unimanual task. Participants performed a total of 18 trials, comprised of four bar end orientation conditions in the 90° and two bar orientation conditions in the 180° version, with each trial performed three times. The start orientation of the objects was blocked and counter-balanced across the participants, and the individual conditions were presented in a randomized order. The primary outcome measure was the proportion of trials that participants complied with end-state comfort (ESC_bim_).

Following the bar transport task, participants had to rate the *perceived grasp comfort* for all grips possibly used during the bar transport task (Hughes et al., 2011; Seegelke et al., 2011). For unimanual trials, participants were informed of the grasp posture (thumb-up, thumb-down, palm-up, and palm-down), after which they reached out and grasped the bar with the preferred hand, held that position for 5 s, then provided a rating of grasp comfort on a scale ranging from zero (very uncomfortable) to 10 (very comfortable). Instructions were similar for bimanual trials, with the exception that participants provided separate comfort ratings for the two grasp postures. Participants performed two trials per condition, and conditions were presented in randomized order. The primary dependent variable was the average comfort rating for trials that included palm-up and thumb-down grasp postures (CR_noESC_, i.e., postures that are seen as uncomfortable due to extreme joint angles; Rosenbaum et al., 1990, 2014; Stöckel et al., 2012). Higher CR_noESC_ values indicate that a posture is perceived as being more comfortable (i.e., extreme joint angles are better tolerated).

Joint flexibility in the shoulder was assessed using the *Back Scratch Test* (Rikli and Jones, 1999, 2013). Participants were asked to place one hand overhead and reach behind the neck to touch down the middle of the back with fingers extended as far as possible. The participant then placed their other hand on the lower back (with the palm facing out) and reached up as far as possible in an attempt to touch or overlap the extended middle fingers of both hands. The experimenter then measured the distance (in cm) between the fingertips. Positive values indicate an overlap (i.e., higher values indicate greater shoulder flexibility). Participants performed two practice and two test trials for each hand. The distance averaged across right and left hand test trials (in cm) was used as measure of shoulder flexibility.

The *Purdue Pegboard Test* (#32020, Lafayette Instruments, Lafayette, IN, United States) was used to assess participant’s gross and fine hand motor skills (i.e., types of manual dexterity). The Purdue Pegboard consisted of two parallel rows of 25 holes in each row, and four concave cups at the top end of the board that held 50 pins (25 pins in the leftmost and rightmost cups), 25 washers (second cup from the left) and 25 collars (third cup from the left). Following the original procedure (Tiffin and Asher, 1948), participants were asked to place as many pins in the row closest to the cup holding the pins, starting with the top hole, in a 30 s time period. Participants first performed the task with the right hand, the left hand, and then with both hands. The scores of the three conditions were combined and used as measure of gross motor skill (*PB_gross_*). Subsequently, participants performed a bimanual assembly task, in which they had to assemble as many pins, collars, and washers as possible in 60 s. Instructions emphasized that both hands should be moving at all times and that the hands should be alternating (i.e., pick up a pin with the right hand, pick up a washer with the left hand, pick up a collar with the right hand, etc.). Participants performed three trials of the bimanual assembly task. The average number of complete assemblies, plus additional parts properly placed at the end of the minute, was used as measure of fine hand motor skill (*PB_fine_*).

#### Cognitive Functions

Cognitive functioning was measured using a comprehensive test battery of well-developed and commonly used tests that specifically addressed the following core executive functions: working memory, inhibitory control and cognitive flexibility and the higher-order executive function response planning and problem-solving (as outlined by Diamond, 2013). The experiments were built and run using the Psychology Experiment Building Language (PEBL) software (Mueller and Piper, 2014; see Piper et al., 2012 for validation).

The *Corsi Block-Tapping Test* (Corsi, 1972; Kessels et al., 2000) was used to assess visuospatial working memory capacity (i.e., the visual-spatial sketchpad; Baddeley, 2003). At the start of each trial, blue-colored blocks were arranged in a static spatial array on the screen with a black background. The blocks changed color from blue to yellow in a predetermined sequence, after which participants had to reproduce the sequence by tapping on the blocks in the same order they were illuminated. The experiment started with sequences of three target blocks and increased by one block (up to a maximum sequence length of nine) as long as the participant correctly reproduced one of the two prior trials. Two non-identical trials were administered for each span-length, regardless of accuracy on the first trial. The test was discontinued when two trials of a given span length were failed. The primary outcome variable was CBT_memoryspan_, which was defined as the maximum sequence length that resulted in correct recall in 50% of trials.

The *Backward Digit Span Task* was used to assess verbal working memory capacity (i.e., the phonological loop; Baddeley, 2003; Conway et al., 2005). At the start of each trial, a sequence of single digits between zero and nine was presented in the middle of a light-gray computer screen (1000 ms inter-stimulus interval). Subsequently, participants used the keyboard to type in the displayed digits in reverse order. Span length ranged from three to 10 digits, with each span-length administered two times. The sequence length was announced prior to each trial and knowledge of results was provided after each trial. If the participant was able to reproduce the sequence correctly in at least one of the two trials for a given span length, then the span was increased by one digit for the next trials. The test was discontinued when the participant failed to reproduce the correct sequence in both trials. The primary outcome measure was the backward digit memory span (Dspan_backward_), which was defined as the longest list that resulted in correct recall in 50% of trials.

The *Flanker task* (FT; Eriksen and Eriksen, 1974; Stins et al., 2007) was used to evaluate inhibitory control and attention. At the start of each trial, a fixation cross was presented for 500 ms, after which five arrows arranged in a horizontal array appeared for 800 ms. Participants were to respond as quickly as possible to the direction the central arrow was pointing to (ignoring the flanking arrows) by pressing the right or left shift key on a standard keyboard. In the congruent condition, the flanking arrows all pointed in the same direction as the target arrow (e.g., “< < < < <”), in the incongruent condition the arrows all pointed in the opposite direction (e.g., “< < > < <”), and in the neutral condition the target arrow was surrounded by two dashes on either side of the arrow (e.g., “- -<- -”) or no distractor at all. Participants were provided with knowledge of results after each trial. In 50% of trials the flanking arrows were in the same direction as the target arrows (congruent), and in 50% of trials the flanking arrows were in the opposite direction as the target arrows (incongruent). There were eight unique conditions comprising the factors condition (congruent, incongruent, neutral 1, neutral 2) and arrow direction (left, right). Each condition was performed 10 times, yielding a total of 80 trials. All trials were fully randomized. Both RT and accuracy information were considered simultaneously (with accuracy information as a covariate) because changes in one variable often result in a change of the other variable (e.g., due to speed-accuracy trade-off; cf. Salthouse, 2010). Thus, mean RT residuals (RT-acc; controlled for accuracy, cf. Salthouse, 2010) were used to measure task-specific processing speed, while the mean RT interference score residuals (RT-acc_interference_; i.e., congruent minus incongruent conditions, cf. Rueda et al., 2005) were used to measure inhibitory control of attention (i.e., selective attention).

The *Simon Task* was used to evaluate inhibitory control at the level of response selection (Lu and Proctor, 1995; Hommel, 2011). At the start of each trial a fixation cross was presented for 400 ms, after which a red or blue circle appeared on the screen. Participants were instructed to press the left key in response to the red circle or the right key in response to the blue key as quickly as possible, regardless of stimulus location (i.e., left or right). Trials in which the stimulus location was on the same side as the required response were congruent, trials in which the stimulus location was on the opposite side as the required response were incongruent, and trials in which the stimulus location was in the middle of the screen were neutral. The experiment consisted of 140 trials (60 congruent, 60 incongruent, 20 neutral) presented in a randomized order. Similar to the Flanker task, RT residuals (RT-acc; controlled for accuracy) averaged across conditions were used to measure task-specific processing speed and the mean RT interference score residuals (RT-acc_interference_; i.e., congruent minus incongruent) were used to measure an individual’s ability to quickly control pre-potent responses during incongruent trials (i.e., response inhibition).

The *Wisconsin Card Sorting Task* (WCST) was used to assess individual’s cognitive flexibility (Grant and Berg, 1948), more specifically their set shifting abilities (Gläscher et al., 2009). This task entails sorting stimulus cards onto one of four piles by matching the color, shape, or number of symbols on the cards. Participants are not informed about the classification rule, but they are given feedback after each attempt (“correct” or “incorrect”) regarding whether or not the respective card was classified according to the current rule. After sorting the cards correctly 10 consecutive times, the classification rule changes. Testing continues until all 128 cards are sorted. The primary outcome measures included the number of perseverative errors (i.e., number of errors in which the participant used the same rule as in the previous trial) which provides a general measure of an individuals’ ability to flexibly adapt to a new rule (or give up an old rule), and non-perseverative errors (i.e., all other errors) which measures an individuals’ ability to stick to a predefined rule.

The *Tower of London task* (TOL) was administered to assess response planning and problem-solving abilities (Shallice, 1982; Anderson et al., 1996). In this task, participants had to rearrange a pile of disks from their original configuration to match the configuration shown at the top of the computer screen. Participants were told that they could move only one disk at a time, and that they could not move a disk onto a pile that has no more room. Further, participants were instructed to try to solve the task in as few steps as possible. The stimuli were based on the standard set of 12 problems (Shallice, 1982) that consisted of 3 disks and constrained pile heights (1, 2, 3). The primary outcome measures included the percentage of trials with perfect solutions (TOL_percentsuccess_, i.e., trials solved in the minimum number of moves) and the time needed until first move for each problem (TOL_firstmove_).

The *Trail Making Test (*TMT; Corrigan and Hinkeldey, 1987; Reitan and Wolfson, 1995; Bowie and Harvey, 2006) was used to assess visual attention (Trails A) and cognitive flexibility (Trails B), more specifically response shifting abilities (Gläscher et al., 2009). Both parts of the TMT involve 25 circles dispersed over the paper in a semi-random order while avoiding any overlap of lines that connect sequential number or letters (cf. Bowie and Harvey, 2006). In Trails Part A (TMT-A), the circles are numbered and the participant is required to draw lines to attach the numbers in numerical order. In Trails Part B (TMT-B), the circles contain both numbers and letters, and the participant has to draw lines to join the circles in ascending fashion, but with the added task of alternating between the number and letters (i.e., 1-A-2-B-3-C, etc.). Participants were instructed to perform the task as accurately and quickly as possible. When an error was made, the participant was instructed to return to the “circle” where the error originated and continue with the task. Dependent variables included time to complete parts A (TMT-A) and B (TMT-B), as well as the B minus A difference (TMT_diff_) as measures of processing speed (TMT-A) and response shifting abilities (TMT_diff_).

### Data Analysis

Preliminary analyses were conducted on all measures of interest to check for normality, sphericity (Mauchly test), univariate and multivariate outliers, with no serious violations noted. Data were collapsed across gender and handedness, as preliminary data analysis did not reveal any systematic differences due to handedness (left-handed, right-handed) or gender (male, female). To control for problems of multiple significance testing (e.g., false discovery rate) in correlation and regression analyses a Benjamini–Hochberg Procedure was applied to the data (Benjamini and Hochberg, 1995). All statistical significant correlations (Pearson r) are reported along with their n-adjusted 95% confidence intervals to take the small sample sizes for sub-groups (e.g., young vs. older adults) into consideration when it comes to the interpretation of correlation coefficients.

## Results

### Age-Related Changes in Cognitive and Motor Abilities

#### Anticipatory Motor Planning Performance

Potential differences in anticipatory motor planning were examined using a mixed-factor Analysis of Variance (ANOVA) with Condition (unimanual vs. bimanual) and Rotation (90° vs. 180°) as within-subjects factors and Age Group (young vs. old adults) as the between-subjects factor. In general, participants selected initial grasp postures that ensured end-state comfort in 78.9% of all trials. Analysis revealed that end-state comfort satisfaction was higher for the young group compared to the old group (73.4% vs. 84.1%), *F*(1,75) = 15.84, *p* < 0.001, η^2^ = 0.17. In addition, end-state comfort was higher when manipulating a single object compared to manipulating two objects (unimanual = 82.6%, bimanual = 74.9%, [*F*(1,75) = 10.18, *p* = 0.002, η^2^ = 0.12]), and for trials that required 90° rotations compared to 180° rotations (90.1% vs. 67.4%, [*F*(1,75) = 119.05, *p* < 0.001, η^2^ = 0.61]). The interaction between condition and rotation was significant, *F*(1,75) = 19.49, *p* < 0.001, η^2^ = 0.21. For trials requiring 180° rotations, end-state comfort values were higher for unimanual compared to bimanual trials (75.1% vs. 59.7%). In contrast, for trials requiring 90° rotations, end-state comfort values were similar regardless of condition (unimanual = 90.0%, bimanual = 90.2%). Moreover, it was found that end-state comfort values were similar for both groups in unimanual conditions (young adults = 85.8%, older adults = 79.3%) but lower for older adults in bimanual conditions (young adults = 82.4%, older adults = 67.5%). This interaction, however, failed to reach statistical significance, *F*(1,75) = 3.07, *p* = 0.08, η^2^ = 0.04. Finally, we separated the older adults in young-olds (61–70 years, *n* = 15) and old-olds (71 years and older, *n* = 22) as done in our previous work (Wunsch et al., 2017) and ran a mixed-factor ANOVA with Condition (unimanual vs. bimanual) as within-subjects factor and Age Group (young vs. young-olds vs. old-olds) as the between-subjects factor. Analysis revealed a significant Age Group effect driven by the bimanual planning condition (87.4% vs. 82.2% vs. 66.2%, [*F*(2,74) = 7.79, *p* = 0.001, η^2^ = 0.17]). *Post hoc* analysis confirmed the difference between young adults and old-olds (*p* < 0.001) as well as between young-olds and old-olds (*p* = 0.001) to be significant, confirming previous work (Wunsch et al., 2017) showing a pronounced age-related decline of anticipatory motor planning skills beyond the age of 70 years.

Furthermore, these results are congruent with prior research demonstrating that the ability to plan ones’ grasp postures is reduced when the task requires two hands (Hughes and Franz, 2008; Logan and Fischman, 2011), which is attributed to greater cognitive demand requirements during the planning of bimanual movements. Given this result, as well as the observation that group differences were observed only for the bimanual condition, only data from bimanual conditions was used in follow-up analyses (e.g., correlation and regression analyses).

#### Motor Functioning

To examine the equality of variance between the two age groups, *F*-tests were conducted separately for each motor skill component of interest. Means and standard deviations for all motor skill measures are displayed in **Table 1**. As with anticipatory motor planning, there were significant differences between groups for almost all motor skill aspects including shoulder flexibility, fine and gross motor dexterity and perceived grasp comfort during bimanual conditions. Ratings of perceived comfort during unimanual conditions were similar for both groups.

**Table 1:** Demographic subject characteristics, motor planning performance, and cognitive and motor functioning of healthy young (*n* = 40) and older adults (*n* = 37).

	Young adults	Older adults	*p*	*F*	η^2^
Age, years	23.13 (2.55)	73.49 (7.12)	<0.001		
Handedness, EHI score	74.22 (28.17)	78.57 (30.24)	0.515		
Physical activity, h/week	9.18 (2.61)	6.35 (5.03)	0.003		
PC use, h/week	13.15 (6.50)	3.28 (7.01)	<0.001		
Video-gaming, h/week	1.48 (2.45)	0.94 (3.38)	0.582		
**Anticipatory motor planning**					
Unimanual, ESC_uni_, %	85.83 (15.24)	79.28 (17.19)	0.080	3.14	0.04
Bimanual, ESC_bim_, %	82.40 (11.39)	67.45 (18.89)	<0.001	18.05	0.19*
**Motor functions**					
Shoulder flexibility, BS, cm	–0.69 (10.71)	–18.69 (13.20)	<0.001	43.43	0.37*
Gross motor dexterity, PB_gross_	42.97 (4.69)	35.95 (4.87)	<0.001	41.50	0.36*
Fine motor dexterity, PB_fine_	37.93 (5.58)	21.18 (5.52)	<0.001	174.52	0.70*
Comfort rating, no-ESCuni, CR_uni_	5.87 (1.44)	6.41 (2.39)	0.233	1.45	0.02
Comfort rating, no-ESCbim, CR_bim_	5.64 (1.55)	6.76 (2.16)	0.010	7.00	0.09
**Cognitive functions**					
*Working memory capacity*					
DSpan_backward_, memory span	6.05 (1.38)	4.06 (1.00)	<0.001	27.44	0.34*
CBT, memory span	5.54 (0.62)	3.97 (1.41)	<0.001	34.33	0.38*
*Inhibitory control, attention and processing speed*					
Flanker, RT-acc, milliseconds	501.17 (75.00)	563.97 (79.12)	0.012	6.75	0.11
Flanker, RT-acc_interference_, milliseconds	43.95 (67.77)	97.07 (67.79)	0.011	7.02	0.12
Simon, RT-acc, milliseconds	441.99 (89.16)	583.21 (94.18)	<0.001	25.49	0.33*
Simon, RT-acc_interference_, milliseconds	37.13 (38.62)	27.99 (39.92)	0.439	0.61	0.01
TMT-A, seconds	19.42 (4.92)	46.20 (19.42)	<0.001	71.22	0.49*
*Cognitive flexibility*					
WCST, % correct	82.27 (7.20)	60.28 (15.71)	<0.001	51.02	0.49*
WCST, perseverative error, %	11.81 (3.75)	13.35 (12.23)	0.470	0.53	0.01
WCST, non-perseverative error, %	5.92 (4.04)	28.25 (23.41)	<0.001	34.66	0.40*
TMT-B, time, seconds	41.57 (12.35)	102.79 (59.27)	<0.001	40.80	0.35*
TMT_diff_, time, seconds	22.16 (9.96)	56.59 (52.05)	<0.001	16.85	0.18*
*Response planning*					
TOL, success, %	67.05 (16.96)	68.75 (15.27)	0.805	0.06	0.00
TOL, first move time, seconds	10.14 (5.92)	14.61 (9.18)	0.038	4.54	0.08

*Reported are means and standard deviations (in brackets), statistical significance (p) of the group comparison based on the *F*-test and eta squared (η^*2*^) as measure of effect size (^∗^indicates large effects).*

**FIGURE 2:**
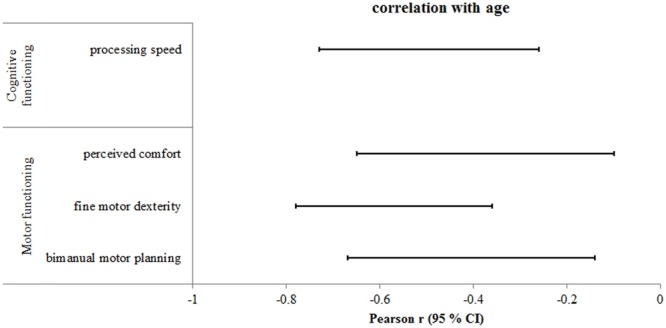
Statistically significant correlations between age and cognitive-motor function measures within the group of older adults. Displayed are the 95% confidence intervals (CI) of the Pearson correlation coefficient (r) adjusted for sample size. Negative correlations indicate a decline of the respective cognitive or motor function with increasing age.

Analyses revealed moderate to very strong negative correlations between chronological age and the following motor functions: bimanual anticipatory motor planning (*r* = - 0.52; 95% CI = -0.66 to -0.33), shoulder flexibility (*r* = -0.63; 95% CI = -0.75 to -0.47), gross motor dexterity (*r* = -0.61; 95% CI = -0.73 to -0.45), and fine motor dexterity (*r* = -0.87; 95% CI = -0.91 to -0.80), all *p*’s < 0.001. When correlation analysis was conducted on only the older adult group, significant negative correlations were observed for the variables bimanual anticipatory motor planning (*r* = -0.44; 95% CI = -0.67 to -0.14), perceived comfort (*r* = -0.41; 95% CI = -0.65 to -0.10), and fine motor dexterity (*r* = -0.61; 95% CI = -0.78 to -0.36), all *p*’s < 0.001 (**Figure 2**). These latter findings indicate that fine motor dexterity, motor planning, and perceived comfort are the aspects of motor functioning most affected by normal aging in individuals over 60 years of age.

#### Cognitive Functioning

*F*-tests were conducted separately for each cognitive function dependent variable of interest in order to examine the equality of variance between the two age groups. As can be seen in **Table 1**, there were significant differences between young and older adult groups for almost all cognitive control processes including working memory, inhibitory control, processing speed, cognitive flexibility, and response planning and problem-solving.

Correlational analysis between chronological age and executive function measures revealed moderate to very strong negative correlations for working memory capacity (DSpan_backward_: *r* = -0.57; 95% CI = -0.73 to -0.36, CBT_memoryspan_: *r* = -0.63; 95% CI = -0.77 to -0.45), inhibitory control (Flanker RT-acc_interference_: *r* = 0.30; 95% CI = 0.03 to 0.53), processing speed (Flanker RT-acc: *r* = 0.31; 95% CI = 0.04 to 0.54, Simon RT-acc: *r* = 0.58; 95% CI = 0.36 to 0.73, TMT-A: *r* = 0.76; 95% CI = 0.64 to 0.84), and both aspects of cognitive flexibility (WCST % correct: *r* = -0.72; 95% CI = -0.83 to -0.56, WCST non-perseverative errors: *r* = 0.64; 95% CI = 0.45 to 0.77, TMT-B: *r* = 0.63; 95% CI = 0.48 to 0.75, TMT_diff_: *r* = 0.45; 95% CI = 0.25 to 0.61). However, when correlational analysis was conducted on only the older adult group, only the relationship between age and processing speed were significantly correlated (TMT-A: *r* = 0.54; 95% CI = 0.26 to 0.73; **Figure 2**). There was no evidence of age-specific decline in executive functions within the group of older adults for any other measures (*r* range = -0.39 to 0.31, all *p*’s > 0.07).

### Overall Associations between Motor Performance and Cognitive Control Processes

Correlational analyses (controlled for age; **Table 2**) revealed that bimanual anticipatory motor planning was positively associated with visuospatial working memory (CBT_memoryspan_: *r* = 0.32, *p* < 0.05), processing speed (Flanker RT-acc: *r* = 0.51, *p* < 0.001), cognitive flexibility (set shifting; WCST % correct: *r* = 0.37, *p* < 0.001, WCST non-perseverative errors: *r* = -0.42, *p* < 0.001), and response planning and problem-solving abilities (TOL_firstmove_: *r* = 0.46, *p* < 0.001). Of these, only Flanker RT-acc, WCST % correct, WCST non-perseverative errors, and TOL first move time yielded a reliably meaningful effect (i.e., at least |*r*|≥ 0.1 with 95% certainty; Cohen, 1988). Regression analysis revealed that anticipatory motor planning performance was significantly predicted by the full model (adjusted *R*^2^ = 0.64, *F*(7,46) = 14.24, *p* < 0.001) which explained 63.6% of the variance in bimanual anticipatory motor planning performance.

**Table 2:** Partial correlations between cognitive and motor function measures controlled for age.

Motor functioning∖ Cognitive functioning	Motor planning (ESC_bim_)	Fine motor dexterity (PB_fine_)	Gross motor dexterity (PB_gross_)	Shoulder flexibility
*Working memory capacity*				
DSpan_backward_, memory span	—	—	—	—
CBT, memory span	0.323^∗^ [0.06, 0.54]	—	–0.279^∗^ [-0.51, -0.01]	—
*Inhibitory control, attention* *and processing speed*
Flanker, RT-acc	**0.511^∗∗∗^ [0.28, 0.69]**	—	–0.309^∗^ [-0.54, -0.04]	—
Flanker, RT-acc_interference_	—	—	—	—
Simon, RT-acc	0.299^∗^ [0.03, 0.53]	—	—	—
Simon, RT-acc_interference_	—	—	—	—
TMT-A, seconds	—	–0.307^∗∗^ [-0.50, -0.08]	—	–0.229^∗^ [-0.44,-0.001]
*Cognitive flexibility*				
WCST, % correct	**0.371^∗∗^ [0.11, 0.59]**	—	–**0.424^∗∗∗^ [**-**0.63,**-**0.17]**	—
WCST, perseverative error, %	—	—	—	—
WCST, non-perseverative error, %	–**0.418^∗∗^ [**-**0.62,**-**0.16]**	—	**0.515^∗∗∗^ [0.28, 0.69]**	—
TMT_diff_, time, seconds	–0.222^∗^ [-0.43, 0.01]	–0.280^∗^ [-0.48, -0.06]	—	—
*Response planning*				
TOL, success, %	—	**0.481^∗∗∗^ [0.12, 0.73]**	—	—
TOL, first move time, seconds	**0.456^∗∗∗^ [0.21, 0.65]**	–0.279^∗^ [-0.51, -0.01]	—	–0.289^∗^ [-0.52, -0.02]

*For all statistically significant correlations Pearson *r-*values are reported along with the 95% confidence interval in brackets. ‘—’ *p* > 0.05, ^∗^*p* < 0.05, ^∗∗^*p* < 0.01, ^∗∗∗^*p* < 0.001; bold-typed values denote reliably meaningful correlations that make at least a small effect with 95% certainty.*

In comparison, fine motor dexterity (PB_fine_) was correlated with processing speed (TMT-A: *r* = -0.31, *p* < 0.01), cognitive flexibility (response shifting; TMT_diff_, *r* = -0.28, *p* < 0.05), and response planning and problem-solving abilities (TOL_firstmove_: *r* = -0.28, *p* < 0.05, TOL_percentsuccess_: *r* = 0.48, *p* < 0.001). Of these significant relationships, only TOL_percentsuccess_ yielded a reliably meaningful effect (i.e., |*r*|≥ 0.1). Regression analysis revealed that fine motor dexterity was significantly predicted by the full model [adjusted *R*^2^ = 0.53, *F*(4,25) = 9.02, *p* < 0.001] explaining 52.5% of the variance in fine motor dexterity. However, only TMT-A performance (processing speed) explained unique portions of fine motor dexterity after controlling for all other variables [β = -0.60, *t*(25) = -4.00, *p* < 0.001].

Gross motor dexterity (PB_gross_) was significantly associated with visuospatial working memory capacity (CBT_memoryspan_: *r* = -0.28, *p* < 0.05), processing speed (Flanker RT-acc: *r* = -0.32, *p* < 0.01), and cognitive flexibility (set shifting; WCST % correct: *r* = -0.42, *p* < 0.001, WCST non-perseverative errors: *r* = -0.52, *p* = 0.001). Of these significant relationships, only the WCST variables yielded a reliably meaningful effect. Regression analysis revealed that 37.2% of the variance in gross motor dexterity was explained by the full model [adjusted *R*^2^ = 0.37, *F*(4,49) = 8.86, *p* < 0.001].

Shoulder flexibility was significantly associated with processing speed (TMT-A: *r* = -0.23, *p* < 0.05), and response planning and problem-solving abilities (TOL_firstmove_: *r* = -0.29, *p* < 0.05). However, none of the measures yielded a reliably meaningful effect.

### Age-Dependent Associations between Anticipatory Motor Planning and Cognitive and Motor Functions

Correlation analyses revealed remarkable differences between young and older adults. In young adults, none of the motor or cognitive function measures were significantly related to motor planning performance. In contrast, motor planning was (statistically meaningful) associated to fine motor dexterity (PB_fine_: *r* = 0.46, *p* < 0.01), perceived grasp comfort (comfort ratings: *r* = 0.45, *p* < 0.001), and response planning times in older adults (TOL first move time, *r* = 0.79, *p* < 0.01; Flanker RT-acc, *r* = 0.73, *p* = 0.001). Fine motor dexterity and response planning times explained 78.6% of the variance in older adults’ motor planning performance [adjusted *R*^2^ = 0.79, *F*(2,11) = 24.93, *p* < 0.001].

## Discussion

In this study age-related decline in anticipatory motor planning was examined as a function of motor (fine and gross motor dexterity, upper extremity flexibility) and cognitive proficiency (working memory, inhibitory control, cognitive flexibility, planning and problem-solving abilities, and processing speed) in a sample of 77 neurologically and physically healthy young and older adults. We also sought to elucidate key (cognitive and motor) factors that are critical for successful motor planning performance in general and in older adults in particular.

### Age-Related Changes in Motor Functioning

Key results showed worse performance on the motor functions bimanual anticipatory motor planning, gross motor dexterity, fine motor dexterity, and shoulder flexibility for older participants compared to young individuals. Considering only individuals aged 60 years and older, chronological age was negatively correlated with the motor functions bimanual anticipatory motor planning, fine motor dexterity, and perceived comfort. Our findings replicate those from previous studies demonstrating age-related decreases in anticipatory motor planning performance (Scharoun et al., 2016; Wunsch et al., 2017) and fine motor dexterity (Desrosiers et al., 1995, 1999; Hoogendam et al., 2014), indicating that these motor skill components are subject to accelerated and progressive decline beyond the age of 60 years. From a physiological perspective, declines in bimanual anticipatory motor planning are thought to arise from alterations in connective tissue (Williams and Goldspink, 1984; James and Parker, 1989), and deteriorations in joint properties (e.g., decrease in synovial viscosity, increases in cartilaginous degeneration, cf. Hall, 1976). In contrast, decreases in fine motor skill performance in the older group likely reflect age-related changes in skeletal muscle structure (e.g., motor unit remodeling, decline in the number of α motor neurons) and function [e.g., motor unit (MU) firing rate variability, reduced sensitivity, cf. Vandervoort, 2002], as well as proprioceptive system (e.g., decreases in the number of joint mechanoreceptors, intrafusal and chain fibers, decrease in muscle spindle sensitivity and diameter, cf. Hughes et al., 2015).

### Age-Related Changes in Cognitive Functioning

Consistent with previous research demonstrating that cognitive capabilities decline as people grow older (cf. Levy, 1994; Park et al., 2003; Hedden and Gabrieli, 2004), the older adult group also exhibited worse performance on measures of working memory, inhibitory control, processing speed, cognitive flexibility, and planning and problem-solving. However, analysis also indicated that only TMT-A scores were significantly correlated with chronological age for individuals in the older adult group, indicating that while a broad range of cognitive functions decline over the lifespan in our sample population, processing speed is the most affected function in individuals beyond 60 years of age. The global slowing of cognitive functions is a characteristic feature of healthy aging (Salthouse, 1996; Fujiyama et al., 2012), and there is debate whether these age-related declines arise from an increase in neural noise (Salthouse, 1996), changes in neural structures and functions (Coxon et al., 2014), and/or a shift in cognitive strategies that prioritize accuracy over speed (Rabbitt, 1979). Irrespective of the exact mechanisms, global cognitive slowing has a significant negative effect on an older person’s ability to perform instrumented activities of daily living (e.g., handling medications, using public transport, housekeeping, managing finances, Barberger-Gateau and Fabrigoule, 1997; Dodge et al., 2006), normal walking (Yogev et al., 2008), climbing stairs (Startzell et al., 2000), and driving performance (cf. Cuenen et al., 2016), which in turn has devastating implications on their functional independence (Royall et al., 2000).

### Associations between Anticipatory Motor Planning and Cognitive Performance

Previous research has reported significant relationships between anticipatory motor planning and working memory (Weigelt et al., 2009; Logan and Fischman, 2011; Stöckel et al., 2012; Stöckel and Hughes, 2016), response planning and problem-solving (Stöckel and Hughes, 2016). In addition to replicating these findings, we also found specific relations between bimanual anticipatory motor planning and the cognitive functions processing speed and cognitive flexibility. In contrast to anticipatory motor planning, the other aspects of motor functioning were not as strongly influenced by cognitive control processes. Regression analyses indicated that cognitive factors explained 64% of the variance in motor planning performance, whereas it explained 53% of the variance in fine motor dexterity, and 37% of gross motor dexterity variance. Thus, the growing corpus of literature indicates that successful motor planning involves specific cognitive processes that allow the individual to consider immediate and future task demands when developing action plans for the two hands, to track performance during execution, and to update the action plan in response to unexpected environmental changes. Furthermore, aging has a detrimental effect on the cognitive processes visuospatial working memory, processing speed, response planning and problem solving, and cognitive flexibility. This cognitive decline in turn impacts the ability of an older individual to appropriately plan their goal-directed grasping movements.

### Implications

At present, 8.9% (650 million) of the worldwide population is aged 65 and older (U.S. Census Bureau, 2013), and is expected to increase to 12 and 16.7% of the total world population in 2030 and 2050, respectively (U.S. Census Bureau, 2013). Moreover, it is expected that the global population of the people aged 80 and older (often termed the “oldest old”) will more than triple between 2015 and 2050, growing from 126.5 million to 446.6 million (He et al., 2015). Given the projected proportions of old people around the world, the results of the present study have important implications for prevention and intervention programs specifically designed to slow cognitive decline and motor impairments in adults over the age of 65 in an effort to maintain anticipatory motor planning skills allowing for an independent living.

Empirical data indicates that (1) physical activity interventions have positive effects on cognitive abilities in healthy normally aging older adults (Colcombe and Kramer, 2003; Voelcker-Rehage et al., 2011), (2) cognitive and physical training programs improve both the targeted cognitive function and processes that were not explicitly trained (Lustig et al., 2009; Zelinski, 2009) and (3) combined cognitive and physical training leads to larger improvements in cognitive function compared to cognitive or physical training alone (Fabre et al., 2002; Oswald et al., 2006; Theill et al., 2013). The results of the present study build on this corpus of work and indicate that prevention and intervention programs should incorporate modules to enable the elderly to regain or maintain processing speed abilities, as this cognitive function likely affects other cognitive and motor processes that require the timely selection of information, decision making, and responses. Specifically, programs that seek to maintain high motor planning abilities, which are important for almost all daily activities and as such for an independent living, should combine modules that have both motor and cognitive demands, as this should maximize benefits at older ages.

### Limitations

Besides the major strengths, the present study also contains some limitations. First, a cross-sectional research design was employed, and as such it is impossible to infer causality between age, motor skill components, and cognitive functioning, nor can individual differences in aging be analyzed. Future research should employ longitudinal designs in order to explain interindividual (between-person) differences, as well as changes that occur within aging individuals (intraindividual differences). Second, the relationship between age and cognition is affected by a variety of factors that differ between countries and communities. Educational attainment (Alley et al., 2007), socioeconomic status (Karlamangla et al., 2009), and race/ethnicity (Clarke et al., 2009; Haas and Rohlfsen, 2010) have been consistently linked to cognitive functioning, and as such future research should include these variables in order to more fully understand how these factors influence cognitive and motor performance during old age.

## Author Contributions

TS and KW developed the study concept, and both of these authors contributed to the design in collaboration with CH. TS collected the data, and analyzed and interpreted it in collaboration with KW and CH. TS, KW, and CH wrote and edited the manuscript. All authors approved the final submitted version of the manuscript.

## Conflict of Interest Statement

The authors declare that the research was conducted in the absence of any commercial or financial relationships that could be construed as a potential conflict of interest. The handling Editor declared a shared affiliation, though no other collaboration, with one of the authors TS and states that the process nevertheless met the standards of a fair and objective review.
